# Object Detection and Scene Perception for Connected and Autonomous Vehicles Using LM-JEPA

**DOI:** 10.3390/s26154894

**Published:** 2026-08-03

**Authors:** Abhishek Gupta, Ajmery Sultana

**Affiliations:** Faculty of Computer Science and Technology, Algoma University, Brampton, ON L6V 1A3, Canada; ajmery.sultana@algomau.ca

**Keywords:** connected and autonomous vehicles, object detection, scene perception, vision–language models, large language models, multi-modal fusion, edge intelligence, real-time inference

## Abstract

**Highlights:**

**What are the main findings?**

- A latent-space LM-JEPA framework enables resource-efficient multi-modal object detection and scene perception for connected and autonomous vehicles, achieving higher perception accuracy with lower inference latency compared to conventional LLM and VLM-based methods.
- Context-aware and adaptive sensor fusion, selective latent transmission, and lightweight edge-assisted reasoning improve cooperative scene understanding, yielding up to 25% better scene understanding, 20% higher intersection success rates, and a 15% reduction in transmitted model parameters.

**What are the implications of the main findings?**

- Latent representation learning provides a practical alternative to token-based LLM and VLM inference, making real-time multi-modal perception feasible on resource-constrained edge devices in connected and autonomous vehicles.
- The proposed framework demonstrates that adaptive latent communication and collaborative reasoning can enhance the scalability, energy efficiency, and safety of future intelligent transportation and cooperative autonomous driving systems.

**Abstract:**

This paper presents the latent model-joint embedding predictive architecture (LM-JEPA), a resource-efficient collaborative perception framework for connected and autonomous vehicles that integrates latent predictive representation learning with lightweight multi-modal reasoning. Autonomous driving in urban and highway environments requires accurate scene understanding under strict latency, energy, and communication constraints, limiting the practicality of large language model (LLM) and vision–language model (VLM)-based approaches in edge deployments. To address this, LM-JEPA encodes heterogeneous inputs including camera, LiDAR, radar, and map data into a unified latent space using a joint embedding predictive architecture, enabling efficient perception and reasoning without token-level inference. Unlike existing latent-space learning approaches that primarily learn predictive visual embeddings for single-modal perception, the proposed framework integrates multi-modal latent reasoning and adaptive sensor fusion to support collaborative perception under resource-constrained vehicular edge environments. The collaborative perception framework introduces a context-adaptive multi-modal fusion mechanism that dynamically weights sensor and model contributions, along with selective latent transmission and adaptive decoding for resource-aware operation. A lightweight VLM is integrated with an edge-assisted vehicular pipeline to support real-time on-vehicle inference with adaptive offloading based on latency and energy constraints, while a latent-space reasoning module enables cooperative decision-making. Experiments on BDD100K and nuScenes-QA show that LM-JEPA improves perception accuracy by 5% and reduces latency by approximately 7% over LLM and VLM baselines, while achieving up to 25% improvement in scene understanding, 20% higher intersection success rates, improved highway merging, and approximately 15% reduction in the transmitted model parameters.

## 1. Introduction

Sixth generation (6G) vehicular networks are characterized by increasing data rates and stringent low-latency requirements, supporting emerging applications such as holographic communications, real-time mapping, and cooperative autonomous driving [1]. Existing perception frameworks enable vehicles to detect and track dynamic obstacles, interpret traffic conditions, and understand road geometry in real time [2]. When combined with edge-deployed large language models (LLMs), these architectures incorporate contextual information to support multi-agent reasoning, predicting behavior of surrounding vehicles, pedestrians, and cyclists and facilitating informed decision-making [3]. However, existing LLM and vision–language model (VLM)-based approaches are prone to unreliable outputs pertaining to hallucinations and sycophancy and have limited ability to jointly perform multi-modal perception, trajectory prediction, and scene-aware reasoning when subject to energy and latency constraints [4]. Latent model-joint embedding predictive architecture (LM-JEPA) builds on the standard JEPA framework by using it to learn compact, predictive latent representations from visual inputs while augmenting it with adaptive decoding, context-aware multi-modal aggregation, and edge-assisted reasoning with selective offloading [5].

### 1.1. Multi-Modal Perception and Vision–Language Models

Recent advances in three-dimensional (3D) object detection have improved geometric perception through feature enhancement and data augmentation, achieving strong performance on benchmark datasets [6]. Multi-modal fusion approaches that combine light detection and ranging (LiDAR) and camera inputs demonstrate the benefits of integrating global and local features for robust detection [7]. However, these methods focus on improving detection accuracy and geometric representation, without incorporating semantic reasoning or natural language understanding for scene interpretation [8]. Although these approaches demonstrate reduction in computational complexity and improvement in detection performance, they do not consider integration with LLM-based semantic reasoning.

Other recent works have enhanced autonomous driving decision-making using retrieval-augmented frameworks and LLMs to improve spatial reasoning and mitigate hallucinations [9]. However, they primarily focus on high-level decision outputs and fine-tuning strategies, such as using language-guided reward signals for policy learning [10]. Furthermore, LLMs are primarily trained on textual data using autoregressive next-token prediction objectives [11]. While these methods achieve notable gains in collision reduction and task completion, they typically utilize VLMs during training or as auxiliary modules and do not analyze the runtime computational cost or feasibility of deploying such perception models on embedded vehicular platforms [12]. This encourages the model to learn concepts instead of discrete tokens. Moreover, while recent studies explore parameter-efficient adaptation and retrieval-augmented mechanisms to improve efficiency, challenges related to computational overhead, scalability, and real-time performance still persist [13].

### 1.2. Motivation

Existing autonomous driving frameworks rely on multi-modal perception pipelines that are sensitive to sensor failures and inconsistencies across camera, LiDAR, and radar inputs. While prior work mitigates this through sensor dropout during training, such approaches primarily improve robustness at the input level without addressing limitations in downstream reasoning [14]. Recent vision–language models incorporate textual supervision using datasets such as nuScenes-QA, but they depend on intermediate caption generation and token-level processing, which introduces computational overhead and information bottlenecks [15]. These limitations hinder real-time deployment and reduce fidelity to the underlying scene dynamics. Furthermore, current methods treat perception and reasoning as loosely coupled tasks, leading to suboptimal integration of spatial and semantic information. This is particularly challenging in dynamic driving scenarios, where temporal consistency and structured scene understanding are critical [16]. Moreover, many existing representation learning methods have primarily focused on predictive visual representation learning and self-supervised feature extraction [12]. Although these approaches significantly reduce computational complexity compared with token-based LLM inference, they do not address collaborative multi-modal perception, prompt-guided reasoning, adaptive latent communication, and resource-aware deployment in connected and autonomous vehicles. Consequently, their applicability to dynamic cooperative driving scenarios remains limited.

Furthermore, existing works either leverage LLMs for high-level reasoning without addressing the efficiency of low-level perception or focus on optimizing communication and computation [17]. Some approaches improve generalization in vision–language navigation through LLM-driven data augmentation; however, they primarily target instruction following and navigation tasks rather than object detection or scene perception [18]. Some frameworks combine multi-modal LLMs such as generative pretrained transformer 4 with vision (GPT-4V) with FL and RL to enhance decision-making and system robustness [19]. While these methods consider metrics such as bandwidth, latency, and resource utilization for scene perception, they increase the computational cost of vision-based scene understanding [20]. To improve deployability, several studies investigate model optimization techniques, including pruning and quantization, and lightweight system integration frameworks for edge deployment [21]. While these efforts reduce memory and computational requirements, they are applied to existing architectures without jointly optimizing end-to-end perception and reasoning. Approaches based on graph neural networks, RL, and unmanned aerial vehicle (UAV)-assisted perception improve decision-making and scene-awareness but depend on conventional perception pipelines or increased sensing coverage rather than efficient onboard perception [22].

Recent advances in graph neural networks (GNNs) and transformer architectures have demonstrated remarkable performance in text mining and document analysis [23]. For example, GNN-based text classification has been employed to model semantic relationships between textual entities for fire-door defect inspection [23], while transformer-based frameworks have been developed for automated text analysis and defect monitoring in building inspection applications [24]. These approaches exploit linguistic dependencies and contextual relationships in textual data to improve classification accuracy. However, unlike GNN and transformer-based text analysis methods, which operate exclusively on textual representations, the proposed framework learns predictive latent representations from multi-modal sensory observations and performs context-aware latent fusion on sensor data gathered from multiple vehicles. Furthermore, selective latent communication and adaptive multi-modal fusion significantly reduce communication overhead while preserving semantic consistency, making the framework suitable for real-time autonomous driving.

To address these challenges, this paper proposes an LM-JEPA framework that combines multi-modal latent fusion, adaptive latent transmission, lightweight VLM reasoning, and edge-assisted collaborative perception. The proposed architecture jointly learns predictive visual representations and language-aware reasoning in a latent space. By avoiding explicit token-level generation and leveraging JEPA-based predictive learning, the proposed framework improves robustness, efficiency, and cross-modal alignment for autonomous driving applications [25]. Moreover, while recent studies explore parameter-efficient adaptation and retrieval-augmented mechanisms to improve efficiency, challenges related to computational overhead, scalability, and real-time performance still persist. Table 1 identifies limitations in existing studies, such as the restricted scalability of LLMs, inadequate handling of multi-modal data, and limited integration of intelligent edge processing. We explore LM-JEPA’s potential to enhance scene perception, improving system performance while ensuring energy efficiency.

### 1.3. Contributions

Although deep learning-based object detection approaches have demonstrated promising performance for intelligent transportation applications, most existing studies primarily focus on perception for specific driving tasks [43]. For example, the parking occupancy detection framework presented in [44] employs RetinaNet and region-based convolutional neural networks to detect vacant parking spaces from surveillance imagery. The proposed architecture emphasizes scalable software architecture and eliminates the need for dedicated parking sensors. The proposed method is suitable for static scene analysis and task-specific object detection, where the objective is accurate classification of parking occupancy. In contrast, the proposed LM-JEPA framework addresses predictive latent representation learning, context-aware multi-modal fusion, and lightweight vision–language reasoning for dynamic traffic environments. Rather than relying solely on supervised object detection, the proposed approach learns compact predictive latent representations that support collaborative perception among connected and autonomous vehicles while reducing communication overhead and computational complexity. The proposed framework extends beyond conventional object detection by jointly enabling semantic scene understanding, temporal prediction, and efficient distributed reasoning for real-time autonomous driving applications. The principal contributions of this work are summarized as follows:

- We propose a novel latent model-joint embedding predictive architecture (LM-JEPA) for connected and autonomous vehicles for object detection, scene perception, and multi-modal reasoning in a compact latent representation. The proposed framework minimizes dependence on computationally intensive token-level inference while supporting efficient real-time operation.
- We develop a context-aware multi-modal perception pipeline that integrates camera, LiDAR, radar, and high-definition (HD) map information through adaptive sensor fusion, selective latent feature transmission, and dynamic edge-assisted inference. The framework enables collaborative perception under stringent latency, communication bandwidth, and energy constraints.
- We perform extensive experimental evaluation on the BDD100K and nuScenes-QA benchmark datasets to assess both perception and scene understanding capabilities. The proposed LM-JEPA consistently outperforms LLM and VLM-based baselines in detection accuracy, reasoning performance, inference latency, and computational efficiency across diverse urban and highway driving scenarios.
- We demonstrate that latent-space predictive learning provides an effective and scalable foundation for next-generation cooperative autonomous driving systems by improving perception reliability, reducing active model complexity, and enabling efficient multi-modal decision-making suitable for resource-constrained vehicular edge platforms.

The rest of this paper is structured as follows: Section 2 presents our system model and formulates the perception and reasoning problem. Section 3 discusses the proposed LM-JEPA reasoning framework. Section 4 discusses the experimental setup and performance evaluation. Section 5 concludes this paper and proposes some avenues for future research.

## 2. System Model

**Figure 1:**
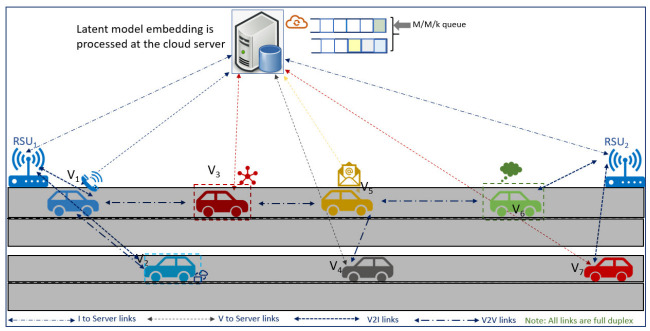
System model: vehicles from different clusters transmit their sensor data to the cloud server, which aggregates them into latent model embeddings and transmits the processed data back to the vehicles.

Figure 1 illustrates our proposed architecture, where a cluster (C) consists of V vehicles $\{v_{1}$, …, $v_{n}\}$. Fusion strategies in multi-modal autonomous perception balance information richness and computational cost. Adaptive modality weights $\omega_{k}$ improve robustness under varying sensing conditions. The language modality provides semantic and reasoning benefits but introduces latency and ambiguity. Let Dr and Dn denote rare and normal driving scenarios, where Dr≪Dn. To address this imbalance, simulation-based augmentation $G_{s}$ is used to expand rare scenario coverage, while prior knowledge $K_{p}$ from foundation models enhances generalization. Let Cm, Mu, and $E_{p}$ denote computational cost, memory usage, and energy consumption. In continual adaptation, new knowledge $K_{n}$ can cause catastrophic forgetting, measured by a forgetting loss $L_{f}$. Regularization $R_{p}$ and experience replay $R_{e}$ mitigate this at additional resource cost. Overall, decision-making is formulated through vision–language–action pipelines that integrate perception with semantic reasoning.

Given sensor observations $S_{t}$, the encoder produces a scene embedding $z_{t}=f_{\theta }\left(S_{t}\right)$ that captures spatial structure, object dynamics, and road context. To enable language-based reasoning, the embedding is projected into a semantic token space via a mapping T(·) and processed by a language model:

(1) $$y=\mathrm{LLM}(T\left(z_{t}\right),q),$$

where *q* is a natural language query and *y* is the generated response. The language model is trained using a cross-entropy objective over answer tokens:

(2) $$L_{\mathrm{QA}}=-\sum_{i}\log P\left(a_{i}|T\left(z_{t}\right),q\right),$$

where $\left\{a_{i}\right\}$ denotes the ground-truth answer sequence. Training is performed using the nuScenes-QA dataset, which provides synchronized camera, LiDAR, radar, and question–answer annotations [45], and the Berkeley DeepDrive (BDD100K) dataset [46], which supports large-scale perception learning. These datasets enable joint optimization of multi-modal representation learning and language-guided reasoning. JEPA is first pretrained to predict masked spatial regions and future latent representations, learning temporally consistent embeddings without explicit reconstruction. The learned embeddings are then aligned with the language model for downstream reasoning tasks. The resulting framework supports anticipatory decision-making by predicting future scene embeddings from observed inputs. For instance, if a pedestrian approaches a crosswalk, the predicted latent state encodes a potential crossing event, enabling the model to generate appropriate responses such as slowing down or stopping. Unlike conventional detect–track–plan pipelines, the proposed approach operates in a unified latent space, integrating perception, prediction, and reasoning. Temporal synchronization and spatial calibration ensure cross-modal consistency, while data normalization and augmentation improve robustness under varying environmental conditions. Table 2 provides a list of key symbols and parameters used in this manuscript.

## 3. LM-JEPA-Based Perception Approach

The proposed LM-JEPA framework models perception as a stochastic process over latent scene representations. Given sensor observations, the encoder produces a sequence of embeddings that evolve over time through predictive updates. This evolution can be interpreted as a finite-state stochastic process, where each state corresponds to a latent representation of the scene. Let Z denote the discrete token space used to represent latent embeddings, and let $Y^{\left(u\right)}$ denote the latent state at iteration *u*. The transition between states is governed by a stochastic operator K, which captures both predictive modeling and uncertainty in dynamic environments. Due to discretization and finite representation, the latent space can be approximated by a finite set Q, enabling the formulation of a transition probability matrix $\prod \in R^{\left|Q|\times |Q\right|}$. Each entry $\pi_{a,b}$ represents the probability of transitioning between latent states under temporal prediction and multi-modal fusion.

The state space is partitioned into optimal representations $Q^{★}$, corresponding to semantically consistent and accurate scene embeddings, and suboptimal representations $Q^{\circ }$. Convergence toward $Q^{★}$ is driven by predictive learning, while stochastic perturbations account for uncertainty and variability in sensor observations. For multiple data samples, parallel processes generate latent states that are aggregated to improve robustness and coverage of the representation space. The convergence behavior is characterized by the optimality gap between the ideal representation and the expected latent state, which decreases over iterations due to improved predictive consistency. This formulation provides a theoretical perspective on the stability and robustness of LM-JEPA, linking predictive representation learning with stochastic state evolution in dynamic driving environments.

### 3.1. Context-Aware Multi-Modal Fusion

The proposed LM-JEPA framework employs a context-aware multi-modal fusion strategy to integrate heterogeneous information obtained from camera images, LiDAR point clouds, radar measurements, HD maps, and vehicle state information. Since individual sensing modalities exhibit different strengths and limitations in varying driving conditions, adaptive fusion enables the perception framework to dynamically exploit the most informative sensor features to maintain robustness in resource-constrained vehicular environments. The multi-modal feature representations are extracted from the individual sensors, where each feature vector is generated by its corresponding modality-specific sensor for latent representation learning. A context vector captures the current driving environment as $c=\left[v,\rho ,\gamma ,\eta ,\kappa \right]$, where *v* denotes the vehicle speed, ρ represents traffic density, γ corresponds to road geometry and lane configuration, ζ denotes environmental conditions such as illumination and weather, and κ represents the relevance of the available sensing modalities in a driving environment. This contextual information characterizes the current driving scenario for the adaptive fusion process. Based on the contextual information, the contribution of each sensing modality is dynamically determined through adaptive weighting as,

(3) $$\alpha_{i}=\frac{\exp\left(g(f_{i},c)\right)}{\sum_{j=1}^{M}\exp\left(g(f_{j},c)\right)},$$

where g(·) denotes a lightweight scoring framework, $\alpha_{i}$ represents the adaptive importance assigned to the $i^{th}$ sensing modality, and *M* denotes the total number of sensing modalities. The fused latent representation is

(4) $$z=\sum_{i=1}^{M}\alpha_{i}f_{i},$$

where z represents the latent embedding forwarded to the LM-JEPA predictor and the subsequent scene reasoning module. Unlike conventional multi-modal fusion approaches that employ fixed feature concatenation or equal-weight feature aggregation, the proposed context-aware fusion mechanism dynamically adjusts sensor contributions according to the prevailing driving conditions. Consequently, relevant sensing modalities are assigned higher importance during perception, while irrelevant or degraded sensor observations are assigned a relatively lower importance to the latent representation.

### 3.2. Latent Representation Learning

Latent model learning in LM-JEPA encodes high-dimensional sensory inputs into compact representations that reduce computational complexity while preserving essential semantics for perception tasks. In the proposed framework, LLMs act as high-level reasoning modules by consuming structured latent embeddings instead of raw sensory inputs. The latent representation serves as an intermediate bridge between perception and reasoning, reducing input complexity and enabling efficient information flow. Each vehicle implements a lightweight VLM for initial feature extraction from sensor data. Let $x_{v}\left(t\right)$ denote the raw sensor measurements collected by vehicle *v* at time *t*. The VLM transforms $x_{v}\left(t\right)$ into a semantic embedding $f_{v}\left(t\right)$:

(5) $$f_{v}\left(t\right)=\mathrm{VLM}\left(x_{v}\left(t\right)\right),$$

where $f_{v}\left(t\right)\in R^{d}$ represents the feature vector capturing both visual and contextual information. These embeddings are processed by a lightweight LLM deployed on the vehicle to perform high-level reasoning, scene understanding, and traffic participant intent inference. The LLM output is denoted as:

(6) $$y_{v}\left(t\right)=\mathrm{LLM}\left(f_{v}\left(t\right),h_{v}\left(t-1\right)\right),$$

where $h_{v}\left(t-1\right)$ encodes the historical context and previous scene annotations, and $y_{v}\left(t\right)$ contains semantic annotations and predicted actions for surrounding agents.

Note, a vehicle computes its own latent representation locally and exchanges only the compact latent embeddings with neighboring vehicles instead of transmitting raw sensor data. Let $N_{v}$ denote the set of neighboring vehicles in the communication range of vehicle *v*. The collaborative latent representation is obtained by aggregating both the local embedding and the received neighboring embeddings according to

(7) $${\tilde{f}}_{v}\left(t\right)=\sum_{u\in N_{v}\cup \left\{v\right\}}\omega_{vu}\left(t\right)\;f_{u}\left(t\right),$$

where $f_{u}\left(t\right)$ is the latent representation generated by vehicle *u*, and $\omega_{vu}\left(t\right)$ denotes the adaptive fusion weight satisfying

(8) $$\sum_{u\in N_{v}\cup \left\{v\right\}}\omega_{vu}\left(t\right)=1,\;\omega_{vu}\left(t\right)\geq 0.$$

The fusion weights indicate the contextual relevance of sensor data gathered from each vehicle. The aggregated latent representation is forwarded to the LLM for semantic reasoning. A vehicle *v* receives latent representations from the set of collaborating vehicles $N_{v}\cup \left\{v\right\}$. For each vehicle *u*, the multi-modal encoder produces a collection of latent features

(9) $$z_{u}\left(t\right)=\left\{z_{u}^{\left(1\right)}\left(t\right),z_{u}^{\left(2\right)}\left(t\right),\ldots ,z_{u}^{\left(M\right)}\left(t\right)\right\},$$

where $z_{u}^{\left(m\right)}\left(t\right)\in R^{d}$ denotes the latent representation of the $m^{th}$ sensing modality. For an input $x_{v}\left(t\right)$, the VLM extracts semantic visual features while the JEPA encoder independently learns a predictive latent representation.

Note, the VLM performs primary visual encoding by processing the raw multi-modal sensor observations and extracting semantic visual features that describe objects, road layouts, traffic conditions, and scene context. These semantic embeddings constitute the initial latent representation used for perception and collaborative information sharing. The JEPA encoder operates on these sensor observations to learn a predictive latent representation of the driving scene. Rather than replacing the VLM encoder or serving as an additional visual feature extractor, JEPA learns compact representations that preserve temporal consistency and predictive information about future driving scenarios. VLM is responsible for semantic perception, whereas JEPA complements this representation by modeling latent predictive dynamics that may not be captured by the visual feature extraction. The latent representations are combined using an adaptive fusion mechanism and are then forwarded to the lightweight LLM for high-level reasoning. VLM focuses on semantic scene understanding, while JEPA captures predictive temporal dynamics and compact latent representations of the driving environment. JEPA is not used as a pretraining stage for the VLM. It serves as a predictive network whose latent representations are adaptively fused with the semantic features extracted by the VLM. Visual encoding is completed before the reasoning stage, while the LLM operates on compact latent representations rather than raw sensory observations. Unlike conventional VLMs, where visual embeddings are directly converted into token sequences and forwarded to an LLM for reasoning, the proposed LM-JEPA framework introduces an intermediate latent fusion stage.

### 3.3. Mapping Continuous Latent Embeddings to the Finite-State Space

**Figure 2:**
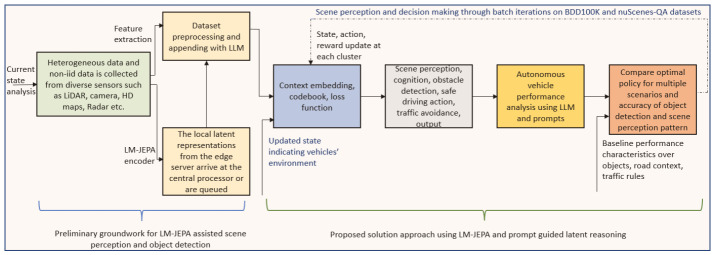
An illustration of the proposed LM-JEPA-based solution approach for object detection and scene perception for connected and autonomous vehicles.

The LM-JEPA encoder produces a continuous latent representation $\in R^{d}$ from multi-modal sensory observations at time *t*. The stochastic perception model is formulated in a finite state space, and the continuous latent vectors are discretized through latent quantization. A codebook $C=\{c_{1},c_{2},\ldots ,c_{K}\}$ is constructed during training using clustering over the latent representations. Each latent vector is mapped to its nearest codeword according to

(10) $$s_{t}=\arg\min_{k\in \{1,\ldots ,K\}}{∥z_{t}-c_{k}∥}_{2},$$

where $s_{t}\in \left\{1,\ldots ,K\right\}$ denotes the discrete perception state associated with the current observation. Here, during network optimization, the LM-JEPA encoder is trained entirely in the continuous latent space using the predictive objective. The latent quantization step maps the learned continuous representations into discrete perception states for stochastic modeling and decision-making. Then, backpropagation is performed through the continuous latent representations generated by the encoder and does not require gradients to propagate through the discrete codebook indices produced by the nearest-neighbor assignment. Each codeword represents a semantic region of the latent space corresponding to similar traffic scenes, object configurations, and environmental contexts. The semantic structure is learned by the continuous JEPA latent space and enables stochastic analysis and decision-making for available perception states. The predictive objective is minimized using backpropagation. Let L denote the training loss and θ represent the trainable network parameters. The parameter update is computed as

(11) $$\theta \leftarrow \theta -\eta \nabla_{\theta }L,$$

where η is the learning rate. Figure 2 illustrates the proposed LM-JEPA-based solution approach for object detection and scene perception for connected and autonomous vehicles.

### 3.4. Prompts for Scene Perception and 3D Reconstruction in Autonomous Driving

We utilize a set of structured prompts designed to evaluate perception, reasoning, and reconstruction capabilities in autonomous driving scenarios for efficient inference over semantic and spatial properties. JEPA learns predictive latent representations that capture high-level scene structure from multi-sensor inputs, while LLMs enable natural language-based reasoning over these representations. In this paper, LM-JEPA learns in a compact latent space where high-level semantic reasoning is facilitated through a lightweight prompt-guided reasoning module. To reduce computational complexity, in this work, we do not automatically construct prompts from the multi-modal perception outputs generated by the LM-JEPA encoder. Rather, we reply on manually designed natural language prompts. The prompts are designed in such a way that they aggregate contextual information extracted from heterogeneous sensing modalities, including camera images, LiDAR point clouds, radar measurements, HD maps, and vehicle state information. The prompts contain semantic descriptions of the current traffic scene, detected objects, spatial relationships, lane topology, traffic signals, surrounding vehicle dynamics, and navigation objectives. A lightweight reasoning module combines latent scene representations with semantic contextual information to infer driving decisions and scene understanding outputs. The prompts offer semantic guidance for latent-space reasoning while avoiding computationally expensive token-level processing. This design enables efficient scene interpretation and cooperative decision-making suitable for real-time deployment in resource-constrained connected and autonomous vehicles.

#### 3.4.1. Prompts for BDD100K

The following prompts focus on perception and short-term prediction tasks:

- Identify all vehicles in the current frame and estimate their relative distances to the ego vehicle.
- Determine whether a pedestrian is approaching the crosswalk ahead.
- Detect traffic lights and identify their current state.
- Evaluate lane boundary structure and determine whether the ego vehicle is centered in its lane.
- Predict whether any vehicle will perform a lane change in the next few seconds.
- Identify potentially occluded objects that may emerge from behind parked vehicles.
- Estimate the speed and trajectory of the vehicle directly ahead of the ego vehicle.

#### 3.4.2. Prompts for nuScenesQA

These prompts evaluate multi-modal reasoning over structured driving scenarios:

- How many pedestrians are present in the scene, and what are their motion directions?
- Is a cyclist approaching from the right side of the ego vehicle?
- Which object is closest to the ego vehicle?
- Which traffic infrastructure elements are visible (e.g., traffic lights, road signs)?
- Is any object likely to cross the road in the next 10 s?
- What is the safest maneuver for the ego vehicle given the current scene context?

#### 3.4.3. Prompts for 3D Reconstruction

In addition to perception and reasoning, we evaluate the model’s learned world representation through spatial prediction tasks under partial observability using the following prompts:

- Predict a latent-consistent 3D reconstruction of the scene from the preceding 5 s of video.
- Infer the 3D geometry of surrounding buildings and infrastructure from multi-modal inputs comprising camera and LiDAR, including occluded regions.
- Predict dense depth maps for visible and partially occluded objects inside a 50-m radius.
- Reconstruct the 3D trajectories of surrounding agents from past observations, including temporally unobserved segments.
- Estimate the spatial layout of roads, sidewalks, curbs, and lane structures from incomplete sensory input.

## 4. Results and Discussion

This section evaluates LM-JEPA for perception and reasoning efficiency on BDD100K and nuScenes-QA datasets. We use the CARLA simulator for vehicle dynamics and process the datasets using an Amazon EC-2 instance. We assume a Manhattan mobility model and vary the number of vehicles from 1 to 100. We compare our results against benchmark LLM, VLM, and multi-modal transformer baselines under identical evaluation settings.

### 4.1. Experimental Setup

The proposed LM-JEPA framework was implemented using Python (version 3.10.13, Python Software Foundation, Wilmington, DE, USA) and TensorFlow (version 2.15.0, Google LLC, Mountain View, CA, USA). Model development and preprocessing were carried out on a laptop equipped with an Intel Core i7 processor and 8 GB RAM running the Ubuntu Linux operating system. Due to the large scale of the BDD100K and nuScenes-QA datasets, model training and performance evaluation were performed on an Amazon EC2 cloud computing instance with graphics processing unit (GPU) acceleration. The proposed LM-JEPA employs a lightweight Vision Transformer (ViT)-based encoder to extract multi-modal latent representations from camera, LiDAR, radar, and HD map inputs. A multi-layer perceptron (MLP) decoder is used during training to optimize the latent predictive objective by mapping latent representations to predictions. The shared latent embedding dimension is fixed at d=512 for all modalities. The visual encoder is initialized using pretrained weights, whereas the predictor, decoder, multi-modal fusion module, and the reasoning network are trained end-to-end on the datasets. The LM-JEPA model was trained using the AdamW optimizer with an initial learning rate of $1\times 10^{-4}$ and a weight decay of $1\times 10^{-4}$. A batch size of 32 was employed, and the learning rate was varied using a cosine annealing schedule. The models were trained for 100 epochs using an input image resolution of 640×640. The simulation parameters and hyperparameter settings are summarized in Table 3. During inference, a batch size of 1 was used, and the latency corresponds to the average inference time measured over the complete test set.

The baseline models were selected to compare with state-of-the-art perception and multi-modal reasoning approaches. GPT-4o and LLaMA-3.1 were selected to represent recent LLM-based multi-modal reasoning frameworks, while Qwen2.5-VL and InternVL2.5 were selected as VLMs because of their recent performance improvements on image understanding and scene interpretation benchmarks. Conventional JEPA models were incorporated to evaluate the advantages of the proposed LM-JEPA over latent predictive representation learning approaches with context-aware multi-modal fusion and latent-space reasoning mechanisms. These baselines cover existing perception methods, including LLM-based reasoning, VLM-based perception, and latent representation learning. However, unlike existing works, the proposed framework in this work is evaluated on the BDD100K and nuScenes-QA benchmark datasets. We evaluate the model in a closed-loop setting within CARLA, where the agent’s actions influence future environment states. All metrics are computed from simulator-provided signals, including vehicle state (position, velocity, acceleration) and event sensors (e.g., collision detection). The reported metrics capture safety, comfort, and control stability:

- Coll: Number of collisions per episode
- SpdVar: Variance of vehicle speed (m/s)^2^, reflecting driving smoothness
- Jerk: Time derivative of acceleration (m/s^3^), measuring control stability
- Route Completion: Fraction of the route successfully traversed

### 4.2. Evaluation Metrics

For object detection on the BDD100K dataset, the primary evaluation metric is the mean average precision (mAP), which measures the average detection precision over different recall levels. Precision and recall quantify the correctness and completeness of object detection results. For scene understanding and question answering on the nuScenes-QA dataset, the scene understanding score is defined as the percentage of correctly interpreted traffic scenes and question–answer pairs. Representation accuracy measures the agreement between the learned latent representations and their corresponding semantic labels. The hallucination score quantifies the proportion of generated semantic descriptions or predicted objects that are not supported by the corresponding sensor observations. Lower hallucination scores indicate better semantic consistency between the predicted output and the observed environment. The planning score evaluates the effectiveness of the generated driving decisions by considering successful navigation, collision avoidance, lane-keeping performance, and compliance with traffic rules under the evaluated driving scenarios. Inference latency is measured as the average end-to-end processing time required to generate a perception and reasoning output from a single multi-modal input and corresponding latent reasoning.

### 4.3. Object Detection and Scene Perception Performance

**Figure 3:**
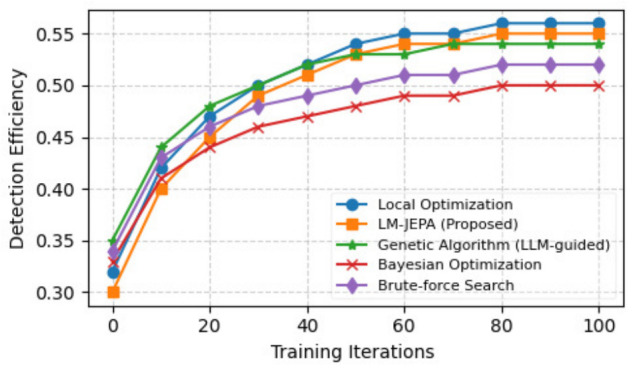
LM-JEPA object detection and scene perception performance.

**Figure 4:**
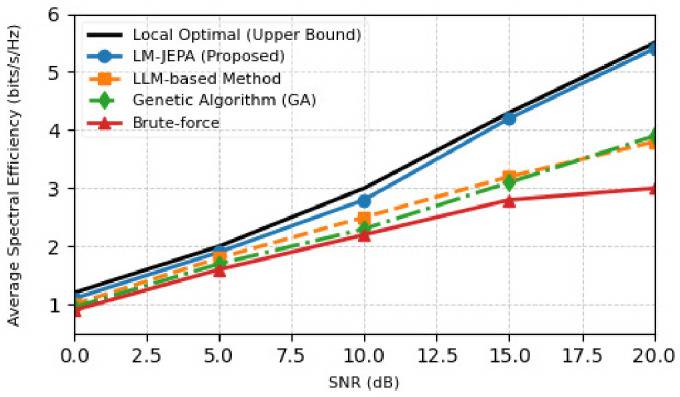
LM-JEPA vs. baseline models: object detection performance.

Figure 3 shows that LM-JEPA maintains stable detection and perception performance under challenging conditions, including occlusion and motion blur. The reduced performance variance indicates improved robustness, attributable to temporally consistent latent representations. Figure 4 illustrates the model performance comparison on object detection tasks using LM-JEPA and baseline models in [8,47]. Note, Figure 3 and Figure 4 illustrate the scene perception performance for a driving scenario where only the compact latent embeddings are exchanged between collaborating vehicles over a wireless communication network. Unlike conventional collaborative perception approaches that transmit raw sensor measurements or high-dimensional feature maps, the proposed LM-JEPA framework communicates compact latent representations. This substantially reduces communication overhead and the corresponding transmission latency. This enables collaborative perception with minimal latency while preserving sufficient semantic information for accurate object detection and scene understanding.

### 4.4. Latency and Computational Efficiency

Table 4 lists the variations in latency and number of trainable parameters. In low-shot settings with only 1% labeled data, performance remains robust, with accuracy ranging from 73.3% to 77.3%, highlighting the efficiency of the learned representations under limited supervision. LM-JEPA also exhibits strong performance on spatial reasoning benchmarks. The model achieves 90% accuracy on counting tasks and 74.6% on distance-based reasoning, demonstrating its ability to encode relational and geometric information in visual scenes. These results suggest that LM-JEPA learns abstract and semantically meaningful features that are well suited for tasks requiring both recognition and structured understanding of spatial relationships. Table 4 demonstrates that LM-JEPA achieves higher accuracy with lower latency and fewer parameters compared to baseline models. This confirms its suitability for real-time deployment. Performance remains stable under low-shot conditions, indicating strong generalization from limited supervision. Additionally, high accuracy in spatial reasoning tasks suggests that LM-JEPA effectively captures geometric relationships. However, limited latency gains imply that further optimization is required for large-scale deployment.

### 4.5. Representation Learning vs. Reasoning Capability

Table 5 highlights the trade-off between reasoning and representation learning. LLM-based methods perform better in structured reasoning tasks, while JEPA excels in spatial representation and low-shot learning. LM-JEPA integrates both paradigms, achieving balanced performance across spatial, temporal, and consistency metrics. In particular, improvements in temporal consistency indicate effective modeling of dynamic scenes. Despite these gains, reasoning performance remains comparable to LLM-only approaches, suggesting further scope for improved integration of symbolic reasoning. Table 5 lists the comparison of LLM-based reasoning and JEPA-based representation learning for autonomous driving perception. JEPA metrics are derived from ImageNet linear probing (79.3–81.1%) and low-shot performance (73.3–77.3%), while spatial reasoning uses CLEVR benchmarks (up to 90.0%). LM-JEPA results match video understanding benchmarks such as Kinetics-400 (81.9%) and Something-Something-v2 (72.2%), capturing temporal consistency.

### 4.6. Driving Task Performance

Table 6 evaluates driving behavior across highway and intersection scenarios. LM-JEPA improves overall driving scores by reducing collision frequency and stabilizing motion dynamics, as reflected in lower jerk and acceleration variance. Notably, performance gains are scenario-dependent. Improvements are more consistent in structured settings (e.g., highway driving), while intersection scenarios remain challenging due to higher uncertainty and interaction complexity. This highlights a remaining limitation in modeling multi-agent dynamics. Latency values remain within real-time constraints, indicating that performance improvements are achieved at a trade-off with computational overhead.

**Figure 5:**
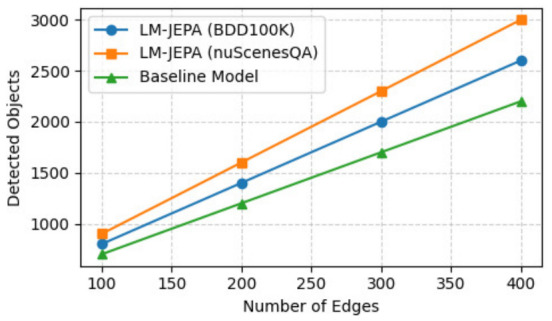
Scene detection accuracy vs. detected objects in LM-JEPA scene perception outputs.

Figure 5 illustrates the relationship between detected objects and scene detection accuracy encompassing number of edges in the LM-JEPA framework. JEPA has strong representation learning capabilities through self-supervised training without reliance on pixel-level reconstruction. On BDD100K, the model achieves linear probing accuracy between 79.3% and 81.1% depending on resolution, indicating that the learned embeddings capture high-level semantic structure. We quantify communication overhead in terms of the total amount of data transmitted from the vehicle to the edge during inference. We measure the effective number of active model parameters during inference. Unlike conventional architectures that utilize all layers uniformly, LM-JEPA employs selective decoding and adaptive layer activation controlled by θ. This reduction translates to fewer active parameters and lower computational overhead without degrading performance. Table 7 presents a comparative evaluation of VLMs, LLMs, and LM-JEPA on the autonomous driving benchmarks BDD100K and nuScenes-QA. The evaluation includes scene reasoning, object counting, and hallucination metrics, assessed in both static and dynamic driving environments.

**Figure 6:**
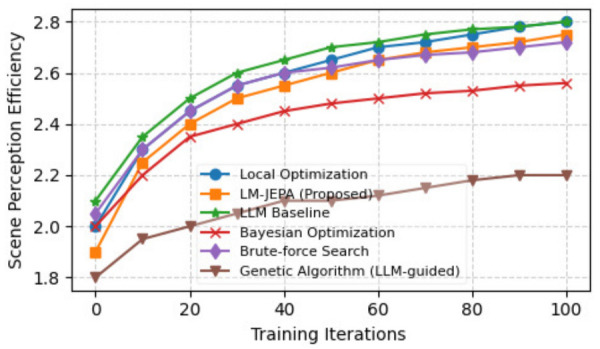
LM-JEPA vs. LLM-based optimization on object detection and scene perception.

Figure 6 illustrates the variation in object detection and scene perception performance for LM-JEPA and LLM-based optimization strategies. JEPA models show higher accuracy than conventional VLMs and LLMs, demonstrating stronger semantic consistency and grounding between visual inputs and text. Comparative analysis shows that while LLM-based approaches perform reasonably well in semantic reasoning, they often fall short in grounded visual understanding. LM-JEPA shows improved performance across both perception and reasoning tasks. JEPA exhibits improved performance at longer time horizons in BDD100K, effectively capturing structured motion patterns and agent interactions. In nuScenes-QA, JEPA outperforms LLMs in next event prediction accuracy, enhancing the connection between visual context and semantic reasoning. LM-JEPA learns latent representations that reflect both spatial structures and temporal evolution, leading to better forecasting accuracy and reduced ambiguity. We assess object detection, motion prediction, and representation quality, reporting metrics such as mean average precision (mAP), motion prediction error (MPE), and representation accuracy (RA). In Figure 6, the number of iterations refers to active processing units, including perception layers, reasoning modules, and communication stages. Increasing the number of iterations corresponds to higher computational complexity and resource usage. However, in resource-constrained edge environments with increasing vehicle density, contention for shared resources leads to higher latency and energy consumption, thereby reducing overall processing throughput. JEPA-based models maintain stable performance under such constraints by leveraging latent-space prediction, which reduces reliance on frequent full-resolution decoding.

Table 8 presents LM-JEPA performance on BDD100K and nuScenes-QA, including perception and reasoning metrics for driving actions such as acceleration, lane changes, and left turns. The reasoning performance on nuScenes-QA is summarized in Table 9. The combined performance evaluation across perception, reasoning, and planning tasks is summarized in Table 10.

Furthermore, LM-JEPA excels in image-level representation tasks, showing improved low-shot generalization and superior performance in dynamic environments. By partitioning temporal embeddings into semantically coherent segments and decoding at representative points, we capture key transitions, like changes in traffic state and agent interactions, while reducing redundant computations.

- **Without temporal prediction**: Removing temporal modeling in JEPA reduces MPE from 1.09 m to 1.35 m.
- **Without multi-modal fusion**: Using only camera images reduces RA from 0.93 to 0.87.
- **Without LLM**: QA accuracy drops from 89.5% to 74.3%, demonstrating the importance of semantic reasoning.

Lower-resolution inputs demonstrate strong transfer performance to higher-resolution scenes, consistently improving both perception accuracy and question-answering metrics. This indicates that multi-modal LLMs capture resolution-invariant semantic features that generalize well across different visual scales. Conversely, models trained on higher-resolution data experience slight degradation when applied to lower-resolution inputs, though their performance remains adequate for tasks such as scene classification and multi-modal reasoning. These findings underscore the adaptability of LLM-based perception modules in managing diverse sensor inputs, which is a significant challenge for large-scale deployment.

**Figure 7:**
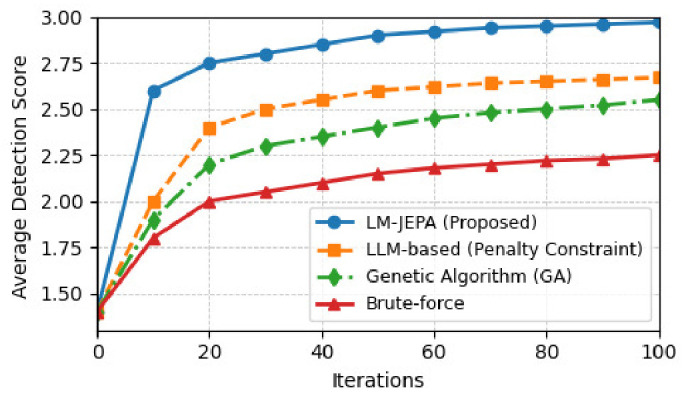
BDD100K and nuScenes-QA performance under resource constraints.

Figure 7 illustrates the performance of models on BDD100K and nuScenes-QA under resource constraints. When combined with JEPA, this framework promotes selective decoding strategies that enhance efficiency without sacrificing accuracy. JEPA-enhanced models exhibit more stable representations and improved resilience to noise, occlusion, and resolution variability across both BDD100K and nuScenes-QA. While high-capacity models provide superior accuracy, their computational costs are a limiting factor. This motivates the exploration of hybrid approaches that balance efficiency and performance. We evaluate communication overhead in terms of transmitted feature size and offloading frequency. In the baseline setting, full-resolution features are transmitted at every time step, resulting in a communication cost of

$$C_{\mathrm{baseline}}=\sum_{t}∥f_{v}\left(t\right)∥.$$

In contrast, LM-JEPA employs selective feature transmission, where only informative latent embeddings are communicated. The resulting communication cost is:

$$C_{\mathrm{JEPA}}=\sum_{t}\delta_{v}\left(t\right)∥{\tilde{f}}_{v}\left(t\right)∥,$$

where ${\tilde{f}}_{v}\left(t\right)$ denotes compressed latent representations.

### 4.7. Vision–Language Alignment and Retrieval Performance

Table 11 compares scene understanding accuracy and cross-modal retrieval performance. LM-JEPA achieves the highest recall (R@1) across both datasets, indicating stronger vision–language alignment. In contrast, LLM-based approaches rely on caption generation, which introduces intermediate bottlenecks and limits fidelity to the underlying visual scene. By directly optimizing joint embeddings, LM-JEPA preserves semantic structure more effectively, leading to substantial gains in retrieval performance. However, small improvements in scene accuracy suggest that enhanced alignment does not fully translate to classification gains. This indicates that retrieval and recognition performance remain partially decoupled. Hence, LM-JEPA-based predictive representation learning improves cross-modal alignment significantly as compared to task-specific classification accuracy.

### 4.8. Model Accuracy and Efficiency Trade-Offs

Table 12 compares model accuracy on BDD100K and nuScenes-QA. JEPA-based models consistently outperform all baselines, with LM-JEPA achieving the highest accuracy across both datasets. These gains are obtained without proportional increases in model size, indicating improved parameter efficiency. Adaptive decoding further enhances efficiency by prioritizing informative regions, reducing redundant computation and inference cost. The performance gains imply effective representation learning rather than only architectural scaling. JEPA-based training produces semantically coherent and noise-resilient embeddings, leading to better robustness under conditions such as occlusion and motion blur. While baseline models benefit from scale, their performance remains limited by indirect visual grounding. In contrast, LM-JEPA directly models visual structure, enabling more reliable perception and improved generalization across driving scenarios.

### 4.9. Perception Task Benchmarking

Table 13 evaluates the detection, segmentation, and tracking performance. Evaluation metrics such as detection, segmentation, tracking, and alignment demonstrate that augmenting standard architectures with LM-JEPA leads to significant gains in performance across different backbone architectures, showing superior results on nuScenes-QA with lower hallucination rates. LM-JEPA achieves the best results across all metrics, with notable reductions in hallucination rates. The improvement in alignment and overall score indicates that JEPA-based training enhances both low-level perception and high-level consistency. Baseline methods reveal comparative detection performance but degrade in alignment and hallucination, highlighting their limitations in maintaining coherent scene representations. Table 13 lists the perception performance on BDD100K across evaluated models. Transitioning to LM-JEPA-based approaches consistently shows performance improvements and reduced hallucinations.

**Figure 8:**
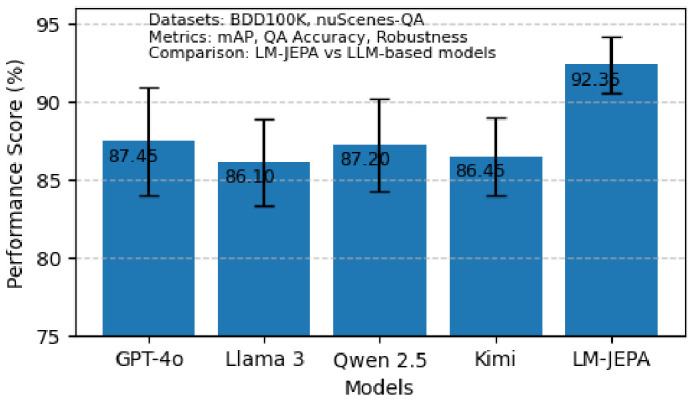
LM-JEPA vs. baseline models.

Figure 8 compares object detection performance across LM-JEPA and baseline models. LM-JEPA consistently outperforms caption-driven VLM pipelines, indicating that direct predictive representation learning preserves richer spatial structure than intermediate textual abstractions. This is advantageous in complex scenes, where caption-based methods suffer from information compression and semantic drift.

### 4.10. Discussion and Limitations

The evaluation presented in this work primarily focuses on standard driving scenarios using the BDD100K and nuScenes-QA benchmark datasets. Although these datasets contain diverse traffic environments, the present work does not explicitly evaluate the proposed LM-JEPA framework under adverse weather conditions such as rain, fog, and snow or under low-light nighttime driving scenarios. Evaluating perception systems under such challenging environmental conditions is critical, as both sensor reliability and scene understanding accuracy can be significantly affected by reduced visibility and environmental disturbances. Consequently, a comprehensive robustness evaluation under varying weather and illumination conditions constitutes an important direction for future research. In the future, the authors aim to investigate the proposed context-aware multi-modal fusion strategy using the weather and illumination subsets available in the BDD100K and nuScenes-QA datasets to further validate the robustness and generalization capability of the proposed LM-JEPA framework.

Moreover, the experimental evaluation assumes reliable communication of latent embeddings among collaborating vehicles and focuses on assessing the perception capability of the proposed LM-JEPA framework. Communication-layer impairments, including packet losses, transmission delays, and queuing delays, are not explicitly modeled in the current experiments. Since the proposed framework exchanges compact latent representations rather than raw sensor data, we conclude that the communication overhead is substantially reduced. However, quantifying the impact of realistic wireless channel degradation and path loss exponents on perception metrics such as mAP and accuracy is an important direction for future work.

## 5. Conclusions

This paper introduced LM-JEPA, a resource-efficient collaborative perception framework for connected and autonomous vehicles that integrates latent predictive representation learning with lightweight multi-modal reasoning. By encoding heterogeneous sensor inputs into a unified latent space, LM-JEPA enables efficient perception and reasoning without relying on token-level inference from conventional LLM-based approaches. The proposed context-adaptive fusion and selective transmission mechanisms support robust performance under latency, energy, and communication constraints. Deployed on the vehicular edge-assisted architecture, LM-JEPA enables real-time inference with adaptive offloading and cooperative decision-making. Evaluations on BDD100K and nuScenes-QA demonstrate consistent improvements in accuracy, latency, and model efficiency over LLM and VLM baselines. These results validate the effectiveness of latent-space integration for scalable, reliable, and resource-aware autonomous driving in complex urban and highway driving scenarios. In the future, we also aim to formulate LM-JEPA-based perception and decision-making strategies where attention-based mechanisms selectively focus on relevant past frames or objects to improve long-term predictions.

## Figures and Tables

**Table 1:** Summary of LLM and VLM-based methods for autonomous driving under resource constraints.

Reference	Problem Addressed	Proposed Solution Mechanism and Identified Gaps
[26]	Limited multi-modal perception and visual grounding in autonomous driving	Text-based reasoning with textual input and generated output; absence of visual understanding limits applicability to scene perception
[27]	Joint visual and language understanding	Multi-modal alignment with visual features and language embeddings; high computational demand under constrained resources
[28]	Lack of explainability and contextual reasoning	Visual question answering using scene input and query; reliance on large-scale infrastructure and limited efficiency
[29]	Handling driving scenarios	Language-guided perception; scalability issues and weak integration with planning modules
[30]	Limited generalization and prediction capability	End-to-end modeling with temporal input and predicted actions; insufficient focus on computational tradeoffs
[31]	VLMs in autonomous vehicles	Task categorization with task groups; lack of integration analysis and real-world constraints
[32]	Need for unified taxonomy of models	Model classification; limited discussion on deployment efficiency
[33]	Reasoning and knowledge integration, unified perception	Knowledge-enhanced models with structured knowledge; lack of VLM implementation
[34]	Perception decision control and error propagation across modules	Multi-modal perception with sensor fusion inputs, localization, object detection, scene understanding, behavior prediction, tracking; weak coordination and limited robustness
[35]	Reasoning and generalization, traffic prediction	Integration of foundation models with VLMs and action generation, forecasting mobility patterns; ignores perception and control tasks, high computational cost and deployment challenges
[36,37]	Mitigating hallucinations in VLMs	Hallucinations in inter-object relationships
[38,39]	Reasoning uncertainty	Visual contrastive decoding for mitigating hallucinations
[40]	Integration of heterogeneous sensor modalities under dynamic conditions	Fusion mapping with adaptive weights; difficulty in accurate weight estimation and real-time stability
[41]	High computational and memory cost	Compression for efficient inference; degradation in rare scenario performance and limited scalability
[42]	Catastrophic forgetting	Regularization and replay with meta learning storage overhead and insufficient scalability for large models

**Table 2:** Main parameter and symbol definitions.

Symbol	Definition
C	Cluster
V, $\{v_{1}$, …, $v_{n}\}$	Vehicles
$\omega_{k}$	Adaptive modality weights
Dn	Normal driving scenarios
Dr	Rare driving scenarios, Dr≪Dn
$G_{s}$	Simulation-based augmentation
$K_{p}$	Prior knowledge from foundation models
Cm	Computational cost
Mu	Memory usage
$E_{p}$	Energy consumption
$K_{n}$	New knowledge
$L_{f}$	Forgetting loss
$R_{p}$	Regularization
$R_{e}$	Experience replay
$S_{t}$	Sensor observations
$z_{t}=f_{\theta }\left(S_{t}\right)$	Scene embedding
T(·)	Semantic token space via a mapping
*q*	Natural language query
*y*	Generated response
$\left\{a_{i}\right\}$	Ground-truth answer sequence
Z	Discrete token space used to represent latent embeddings
$Y^{\left(u\right)}$	Latent state at iteration *u*
K	Stochastic operator
Q	Latent space approximated by a finite set
$\prod \in R^{\left|Q|\times |Q\right|}$	Transition probability matrix
$\pi_{a,b}$	Probability of transitioning between latent states
$Q^{★}$	Optimal representations
$Q^{\circ }$	Suboptimal representations
$x_{v}\left(t\right)$	Raw sensor measurements collected by vehicle *v* at time *t*
$f_{v}\left(t\right)\in R^{d}$	Feature vector capturing both visual and contextual information
*v*	Vehicle speed
ρ	Traffic density
γ	Road geometry and lane configuration
ζ	Environmental conditions, illumination and weather
κ	Relevance of the sensing modalities
g(·)	A lightweight scoring framework
$\alpha_{i}$	Adaptive importance of $i^{th}$ sensing modality
*M*	Total number of sensing modalities
$h_{v}\left(t-1\right)$	Encoding of historical context and previous scene annotations
$y_{v}\left(t\right)$	Semantic annotations and predicted actions for surrounding agents
$N_{v}$	Set of neighboring vehicles in communication range of vehicle *v*
$f_{u}\left(t\right)$	Latent representation generated by vehicle *u*
$\omega_{vu}\left(t\right)$	Adaptive fusion weight
$z_{u}\left(t\right)$	Collection of latent features from vehicle *u*
η	Learning rate in backpropagation
L	Training losses
θ	Trainable network parameters

**Table 3:** Simulation parameters, hardware configuration, and hyperparameter settings.

Parameter	Value
Number of vehicles	1–100
Number of latent tokens	1–8
Edge infrastructure range	100 m–3 km
Urban scene coverage area	4 km × 2 km
Communication frequency	5.9 GHz
Inter-vehicle distance	50–200 m
Perception features per frame	25
Edge node deployment height	10 m–100 m
Road network length	1–4 km
Vehicle speed range	0–100 km/h
Data packet size	1 byte–3 MB
Datasets used	BDD100K, nuScenes-QA
Edge buffer size (B)	1 GB
Edge node transmit power	20 dBm (100 mW)
Receiver sensitivity	−80 dBm
Edge node energy budget	600 kJ
Vehicle transmit power	25 dBm (316.2 mW)
Standard deviation in speed	10 km/h
Development platform	Intel Core i7 Laptop
System RAM	8 GB
Operating system	Ubuntu Linux
Training platform	Amazon EC2 (GPU Instance)
Framework	TensorFlow
Optimizer	AdamW
Learning rate	$1\times 10^{-4}$ to 0.95
Batch size	32
Training epochs	100
Input resolution	640×640
Learning rate scheduler	Cosine Annealing
Encoder	Lightweight Vision Transformer (ViT)
Predictor	Multi-Layer Perceptron (MLP)
Decoder	Two-Layer MLP

**Table 4:** Comparison of LM-JEPA with LLM/VLM baselines on BDD100K perception and nuScenes-QA reasoning tasks.

	BDD100K (Perception)		nuScenes-QA (Reasoning)		
Method	Accuracy (%)	Latency (ms)	Accuracy (%)	Latency (ms)	Params (M)
LLM-Based VLM	78.4	44.7	76.9	44.7	380.2
Vision–Language Model	80.1	42.3	78.5	42.3	350.6
Multi-Modal Transformer	81.7	43.8	80.2	43.8	340.9
LM-JEPA	86.7	41.5	85.3	41.5	325.1

**Table 5:** Comparison of LLM-based reasoning and JEPA-based representation learning for autonomous driving perception, with metrics from ImageNet linear probing, low-shot evaluation, CLEVR, Kinetics-400, and Something-Something-v2.

Dataset/Task	LLM (GPT-Style Reasoning)			JEPA (Image Representation)			LM-JEPA (Video Model)		
	QA Acc.	Parsing	Logic Cov.	Linear Acc.	Low-Shot	Spatial	Action Acc.	Temporal	Consistency
BDD100K: Object Detection	61.2	55.3	58.1	79.3	73.3	74.6	77.9	72.2	75.1
BDD100K: Lane Detection	58.4	52.1	54.7	79.3	73.3	90.0	77.9	72.2	81.5
BDD100K: Drivable Area	60.1	53.7	56.2	81.1	77.3	74.6	77.9	72.2	78.4
BDD100K: Traffic Sign Recognition	64.5	58.6	61.3	81.1	77.3	74.6	77.9	72.2	76.2
nuScenes-QA: Scene Understanding	65.4	59.2	61.8	81.1	77.3	74.6	81.9	72.2	77.9
nuScenes-QA: Spatial Reasoning	62.7	55.6	58.9	79.3	73.3	90.0	81.9	72.2	80.3
nuScenes-QA: Temporal Reasoning	59.8	52.1	55.3	79.3	73.3	74.6	81.9	72.2	82.7
nuScenes-QA: View Consistency	60.9	53.7	56.8	81.1	77.3	74.6	81.9	72.2	83.5
Mean	61.6	55.0	57.9	80.3	75.2	78.5	79.9	72.2	79.5
Median	60.5	53.7	56.5	80.2	75.3	74.6	79.9	72.2	79.3

**Table 6:** LM-JEPA driving performance on BDD100K and nuScenes-QA.

Scen.	Behav.	Cmd	Coll.	SpdVar	Acc	Jerk	Lat.	Score
Hwy	Overtake	I	2.88	5.02	0.24	2.64	1.68	85.05
		II	1.94	4.05	0.24	2.81	1.87	86.12
		III	3.07	1.26	0.18	2.64	1.86	91.12
		Base	3.26	2.91	0.35	2.83	–	80.00
	Follow	I	6.52	0.94	0.15	2.35	1.61	87.10
		II	7.84	1.11	0.05	2.38	1.64	86.23
		III	6.78	1.37	0.09	2.31	1.64	86.26
		Base	4.02	0.78	0.22	2.50	–	86.00
	Right Lane	I	8.77	1.69	0.17	2.39	1.32	90.88
		II	4.54	1.18	0.15	2.44	1.83	91.51
		III	7.29	0.23	0.13	2.61	1.22	92.18
		Base	4.70	7.39	0.22	2.77	–	86.00
Int.	No Yield	I	0.89	0.29	0.26	2.28	1.65	59.67
		II	0.89	0.22	0.28	2.55	1.78	59.60
		III	1.04	0.21	0.26	2.32	1.52	60.32
		Base	1.14	0.46	0.46	2.34	–	56.00
	Yield	I	–	0.29	0.52	2.27	1.47	91.53
		II	–	0.22	0.82	2.54	1.43	89.50
		III	–	0.21	0.48	2.28	1.38	91.92
		Base	–	1.67	0.90	–	–	–

**Table 7:** Evaluation of VLMs, LLMs, and LM-JEPA on autonomous driving benchmarks (BDD100K and nuScenes-QA). Metrics include scene reasoning, object counting, and hallucination (evaluated in static and dynamic environments).

	Scene Reasoning		Object Counting		Hallucination (Static)		Hallucination (Dynamic)	
Dataset	Accuracy	Score	Accuracy	Score	Accuracy	Score	Accuracy	Score
BDD100K	58.7	61.2	62.5	65.1	81.3	84.6	79.8	83.2
nuScenes-QA	54.9	57.6	59.1	61.8	78.5	82.1	76.4	80.7
**Model-wise Highlights (LLM/VLM/JEPA)**								
InternVL-Chat (VLM)	59.5	62.0	63.8	66.4	83.4	86.2	81.7	84.9
Qwen-VL (VLM)	57.3	60.1	62.9	65.0	82.7	85.6	80.9	83.8
LLaMA-3.1 + Vision (LLM)	56.8	59.4	61.2	63.5	80.6	83.9	78.8	82.4
GPT-4o (LLM)	60.9	63.7	65.4	68.2	85.1	88.3	83.5	86.7
JEPA (1.2B)	61.5	64.3	66.9	69.8	85.7	88.9	84.6	87.5
LM-JEPA (1.6B)	64.2	67.1	69.5	72.3	88.6	91.2	87.3	90.1

**Table 8:** LM-JEPA driving performance on BDD100K and nuScenes-QA benchmarks.

Scenario	Model	Coll.	SpdVar	Acc	Jerk	Lat.	Hum.	Scn.	Score
Acceleration	Base	2.44	28.8	0.36	0.78	–	92.0	60.0	75.6
	GPT-4o	2.52	30.8	0.39	0.83	5.82	92.9	71.5	76.4
	LM-JEPA	2.46	30.8	0.39	0.81	1.98	96.3	60.9	76.5
Lane Change	Base	2.44	3.91	1.65	0.37	–	88.5	60.0	74.5
	GPT-4o	2.71	3.88	2.23	0.53	4.84	90.4	88.6	78.4
	LM-JEPA	2.15	4.07	2.15	0.41	1.83	92.2	71.9	77.5
Left Turn	Base	–	1.12	7.52	0.22	–	88.0	60.0	70.4
	GPT-4o	–	0.93	11.5	0.29	5.23	91.3	85.0	71.4
	LM-JEPA	–	0.94	6.74	0.19	1.64	90.2	67.8	74.4

**Table 9:** nuScenes-QA reasoning performance.

Model	Det	Seg	Trk	QA	Hall	Align	Ovrl	Rank
LLaMA3.1+V	0.542	0.471	0.438	0.561	0.261	0.552	0.538	6.8
Qwen2.5-VL	0.557	0.482	0.449	0.574	0.248	0.569	0.551	5.9
GPT-4o	0.589	0.503	0.472	0.612	0.221	0.601	0.583	4.1
JEPA	0.601	0.517	0.486	0.629	0.204	0.618	0.596	3.2
LM-JEPA	0.634	0.541	0.512	0.661	0.178	0.647	0.629	1.9

**Table 10:** Combined performance evaluation across perception, reasoning, and planning tasks.

Model	mAP	MPE	RA	QA	Plan
Standard LLM	45.2	3.82	0.58	61.7	52.1
JEPA-only	87.5	1.15	0.92	74.3	78.6
JEPA + LLM	88.3	1.09	0.93	89.5	91.2

**Table 11:** VLM and LM-JEPA performance on BDD100K and nuScenes-QA (IVL: InternVL2.5 26B, Qwen: Qwen2.5-VL 72B, LLaMA-3.1 70B, GPT-4o, JEPA SFT).

Data	Scene Acc. (%)				R@1 (%)			
	IVL	Qwen	LLaMA	GPT4o	IVL	Qwen	LLaMA	JEPA
BDD100K	61.8	64.5	62.7	66.4	47.8	50.2	48.9	59.8
nuScenes-QA	56.9	59.8	57.4	60.3	43.6	45.7	44.2	54.6

**Table 12:** Model accuracy (%) on BDD100K and nuScenes-QA (IVL: InternVL2.5 38B, Qwen-VL: Qwen2.5-VL 7B, LLaMA-3.1 70B, GPT-4o, Claude-3.5, JEPA, and LM-JEPA).

Data	IVL	Qwen-VL	LLaMA	GPT4o	Claude	JEPA	LM-JEPA
BDD100K	54.1	49.3	53.9	52.7	54.9	60.1	63.5
nuScenes-QA	49.5	44.8	48.7	48.3	50.2	55.8	58.9

**Table 13:** BDD100K perception task performance (detection, segmentation, tracking) for all evaluated models.

Model	Det	Seg	Trk	QA	Hall	Align	Ovrl	Rank
MLP	0.527	0.463	0.412	0.498	0.221	0.531	0.502	9.2
StratLR	0.563	0.491	0.438	0.521	0.204	0.566	0.531	6.4
SwitchEM	0.571	0.498	0.446	0.533	0.198	0.574	0.539	5.9
MinRec	0.552	0.482	0.431	0.515	0.214	0.559	0.522	7.5
SubTab	0.545	0.479	0.429	0.512	0.218	0.553	0.519	8.1
JEPA	0.598	0.521	0.469	0.562	0.183	0.602	0.556	3.8
ResNet	0.551	0.478	0.436	0.514	0.244	0.552	0.529	9.8
FTARL	0.586	0.503	0.459	0.539	0.221	0.584	0.552	6.3
VIME	0.574	0.496	0.452	0.531	0.228	0.571	0.544	7.2
BinRecon	0.561	0.487	0.448	0.526	0.233	0.566	0.538	7.0
SubTab	0.558	0.485	0.446	0.524	0.236	0.563	0.536	7.5
LM-JEPA	0.623	0.537	0.491	0.584	0.198	0.628	0.577	2.6

## Data Availability

The original contributions presented in this study are included in the article. Further inquiries can be directed to the corresponding author.

## Abbreviations

The following abbreviations are used in this manuscript:

3D	Three-dimensional
6G	Sixth generation (communication networks)
BDD100K	Berkeley DeepDrive (Dataset)
GNN	Graph neural network
GPT	Generative pretrained transformer
GPT-4V	Generative pretrained transformer 4 with vision
HD	High-definition
LiDAR	Light detection and ranging
LLM	Large language model
LM-JEPA	Latent model-joint embedding predictive architecture
mAP	Mean average precision
MLP	Multi-layer perceptron
ViT	Vision transformer
VLM	Vision–language model

## References

[1] Khalil R.A., Safelnasr Z., Yemane N., Kedir M., Shafiqurrahman A., Saeed N. (2024). Advanced Learning Technologies for Intelligent Transportation Systems: Prospects and Challenges. IEEE Open J. Veh. Technol..

[2] Cheng X., Liu B., Liu X., Liu E., Huang Z. (2025). Foundation Model Empowered Synesthesia of Machines (SoM): AI-native Intelligent Multi-Modal Sensing-Communication Integration. IEEE Trans. Netw. Sci. Eng..

[3] Zhou H., Hu C., Yuan Y., Cui Y., Jin Y., Chen C., Wu H., Yuan D., Jiang L., Wu D. (2025). Large Language Model (LLM) for Telecommunications: A Comprehensive Survey on Principles, Key Techniques, and Opportunities. IEEE Commun. Surv. Tutor..

[4] Zhou H., Hu C., Yuan D., Yuan Y., Wu D., Chen X., Tabassum H., Liu X. (2025). Large Language Models for Wireless Networks: An Overview from the Prompt Engineering Perspective. IEEE Wirel. Commun..

[5] Ferrag M.A., Lakas A., Tihanyi N., Debbah M. (2026). LLM and AI Agents for Autonomous Systems: A Survey of Applications, Datasets, and Security Challenges. IEEE Open J. Intell. Transp. Syst..

[6] Tian H., Reddy K., Feng Y., Quddus M., Demiris Y., Angeloudis P. (2026). Large (Vision) Language Models for Autonomous Vehicles: Current Trends and Future Directions. IEEE Trans. Intell. Transp. Syst..

[7] Chi F., Wang Y., Nasiopoulos P., Leung V.C. (2025). Multi-Agent Collaborative Decision-Making Using Small Vision-Language Models for Autonomous Driving. IEEE Internet Things J..

[8] Xiong G., Liu S., Yan Y., Li Q., Li H. (2025). Efficacy of Autonomous Vehicle’s Adaptive Decision-Making Based on Large Language Models Across Multiple Driving Scenarios. IEEE Access.

[9] Sharshar A., Khan L.U., Ullah W., Guizani M. (2025). Vision-Language Models for Edge Networks: A Comprehensive Survey. IEEE Internet Things J..

[10] Wang J., Ren H., Zhu X., Ma Z. (2025). Enhancing Autonomous Vehicle Decision-Making Through Policy Transfer With Large Language Model. IEEE Trans. Intell. Transp. Syst..

[11] Zhu Y., Li Y., Li Z., Li Z., Guo G. (2025). Game-Theoretic Decision-Making for Autonomous Vehicles at Unsignalized Intersections under Communication Interferences: A Novel Risk-Adaptive Approach. IEEE Trans. Veh. Technol..

[12] Liu Q., Tang Y., Li X., Du G., Li Z. (2025). Enhancing the Collaborative Decision-Making Performance of Connected and Autonomous Vehicles: A Multi-Modal Failure-Aware Graph Representation Approach. IEEE Trans. Intell. Transp. Syst..

[13] Cui Y., Huang S., Zhong J., Liu Z., Wang Y., Sun C., Li B., Wang X., Khajepour A. (2024). DriveLLM: Charting the Path Toward Full Autonomous Driving With Large Language Models. IEEE Trans. Intell. Veh..

[14] Zheng Y., Xing Z., Zhang Q., Jin B., Li P., Zheng Y., Xia Z., Chen Y., Zhao D. (2026). PlanAgent: A Multi-modal Large Language Agent for Closed-loop Vehicle Motion Planning. IEEE Trans. Cogn. Dev. Syst..

[15] Deng Y., Tu Z., Yao J., Zhang M., Zhang T., Zheng X. (2025). TARGET: Traffic Rule-Based Test Generation for Autonomous Driving via Validated LLM-Guided Knowledge Extraction. IEEE Trans. Softw. Eng..

[16] Noh H., Shim B., Yang H.J. (2025). Adaptive Resource Allocation Optimization Using Large Language Models in Dynamic Wireless Environments. IEEE Trans. Veh. Technol..

[17] Friha O., Amine Ferrag M., Kantarci B., Cakmak B., Ozgun A., Ghoualmi-Zine N. (2024). LLM-Based Edge Intelligence: A Comprehensive Survey on Architectures, Applications, Security and Trustworthiness. IEEE Open J. Commun. Soc..

[18] Gong Y., Zhang X., Lu J., Jiang X., Wang Z., Liu H., Li Z., Wang L., Yang Q., Wu X. (2025). Steering Angle-Guided Multimodal Fusion Lane Detection for Autonomous Driving. IEEE Trans. Intell. Transp. Syst..

[19] Mortlock T., Chen L., Smereka J.M., Khargonekar P., Abdullah Al Faruque M. (2025). Fuse It or Lose It? Analyzing the Effects of Sensor Diversity on Multimodal Ensembles for Autonomous Vehicle Perception. IEEE Trans. Intell. Transp. Syst..

[20] Rafiq M., Sung M., Rafiq G., Sang Choi G. (2025). Camscribe: Enhanced Dashcam Video Descriptions Through Multimodal Spatiotemporal and Object Detection for Autonomous Vehicles. IEEE Access.

[21] Huang S., Shi F., Sun C., Zhong J., Ning M., Yang Y., Lu Y., Wang H., Khajepour A. (2025). DriveSOTIF: Advancing SOTIF Through Multimodal Large Language Models. IEEE Trans. Veh. Technol..

[22] Wei Z., Lin B., Nie Y., Chen J., Ma S., Xu H., Liang X. (2025). Unseen From Seen: Rewriting Observation-Instruction Using Foundation Models for Augmenting Vision-Language Navigation. IEEE Trans. Neural Netw. Learn. Syst..

[23] Wang S. (2025). Graph neural network–driven text classification for fire-door defect inspection in pre-completion construction. Sci. Rep..

[24] Wang S. (2025). Development of an automated transformer-based text analysis framework for monitoring fire door defects in buildings. Sci. Rep..

[25] Hassan M., Kabir M.E., Jusoh M., Ki An H., Negnevitsky M., Li C. (2025). Large Language Models in Transportation: A Comprehensive Bibliometric Analysis of Emerging Trends, Challenges, and Future Research. IEEE Access.

[26] Mohammed A., Kora R. (2025). A Comprehensive Overview and Analysis of Large Language Models: Trends and Challenges. IEEE Access.

[27] McIntosh T.R., Susnjak T., Arachchilage N., Liu T., Xu D., Watters P., Halgamuge M.N. (2026). Inadequacies of Large Language Model Benchmarks in the Era of Generative Artificial Intelligence. IEEE Trans. Artif. Intell..

[28] Luo H., Sun G., Liu Y., Zhao D., Niyato D., Yu H., Dustdar S. (2026). A Weighted Byzantine Fault Tolerance Consensus Driven Trusted Multiple Large Language Models Network. IEEE Trans. Cogn. Commun. Netw..

[29] Lee H., Zhou W., Debbah M., Lee I. (2025). On the Convergence of Large Language Model Optimizer for Black-Box Network Management. IEEE Trans. Commun..

[30] Liu X., Gao S., Liu B., Cheng X., Yang L. (2025). LLM4WM: Adapting LLM for Wireless Multi-Tasking. IEEE Trans. Mach. Learn. Commun. Netw..

[31] Kang J., Ko W., Lee Y., Lee K., Yun I. (2025). Large Language Model-Based Functional Scenario Generation for Automated Vehicle Safety Evaluation Using Vehicle and Pedestrian Traffic Accident Data. IEEE Access.

[32] Li J., Wang Z., Gong D., Wang C. (2025). SCNet3D: Rethinking the Feature Extraction Process of Pillar-Based 3D Object Detection. IEEE Trans. Intell. Transp. Syst..

[33] Xue P., Wu L., Yu Z., Jin Z., Yang Z., Li X., Yang Z., Tan Y. (2024). Automated Commit Message Generation with Large Language Models: An Empirical Study and Beyond. IEEE Trans. Softw. Eng..

[34] Zhao J., Wen T., Cheong K.H. (2026). Can Large Language Models Be Trusted as Evolutionary Optimizers for Network-Structured Combinatorial Problems?. IEEE Trans. Netw. Sci. Eng..

[35] Liu C., Zhao J. (2026). Enhancing Stability and Resource Efficiency in LLM Training for Edge-Assisted Mobile Systems. IEEE Trans. Mob. Comput..

[36] Wu M., Li J., Ji J., Hao F., Sun X., Ji R. (2026). Evaluating and Mitigating Relationship Hallucinations in Large Vision-Language Models. IEEE Trans. Pattern Anal. Mach. Intell..

[37] Qiang P., Tan H., Zhang H., Li X., Li R., Liang J. (2026). Mitigating Hallucinations in Large Vision-Language Models via Visual-Enhanced Contrastive Decoding. IEEE Trans. Multimed..

[38] Li S., Xu X., Meng W., Song J., Peng C., Shen H.T. (2025). Mitigating Hallucinations in Large Vision-Language Models via Reasoning Uncertainty-Guided Refinement. IEEE Trans. Multimed..

[39] Fan J., Wu J., Chu H., Ge Q., Gao B. (2026). Hallucination Elimination and Text Annotation Framework for Large Vision-Language Models in Traffic Scenarios. IEEE Trans. Intell. Transp. Syst..

[40] Dastagir M.B.A., Han D. (2024). Towards Hybrid Quantum-Classical Deep Learning Architecture for Indoor-Outdoor Detection Using QCNN-LSTM and Cluster State Signal Processing. IEEE Signal Process. Lett..

[41] Schafer M., Nadi S., Eghbali A., Tip F. (2024). An Empirical Evaluation of Using Large Language Models for Automated Unit Test Generation. IEEE Trans. Softw. Eng..

[42] Anne T., Syrkis N., Elhosni M., Turati F., Legendre F., Jaquier A., Risi S. (2025). Harnessing Language for Coordination: A Framework and Benchmark for LLM-Driven Multiagent Control. IEEE Trans. Games.

[43] Gupta A., Anpalagan A., Guan L., Khwaja A.S. (2021). Deep learning for object detection and scene perception in self-driving cars: Survey, challenges, and open issues. Array.

[44] Padmasiri H., Madurawe R., Abeysinghe C., Meedeniya D. (2020). Automated Vehicle Parking Occupancy Detection in Real-Time. Proceedings of the 2020 Moratuwa Engineering Research Conference (MERCon), Moratuwa, Sri Lanka, 28–30 July 2020.

[45] Qian T., Chen J., Zhuo L., Jiao Y., Jiang Y.G. (2024). NuScenes-QA: A Multi-Modal Visual Question Answering Benchmark for Autonomous Driving Scenario. Proc. Conf. Artif. Intell..

[46] Yu F., Chen H., Wang X., Xian W., Chen Y., Liu F., Madhavan V., Darrell T. (2020). BDD100K: A Diverse Driving Dataset for Heterogeneous Multitask Learning. Proceedings of the (IEEE Computer Society Conference on Computer Vision and Pattern Recognition. Online).

[47] Mayumu N., Deng X., Bagula A., Khan S.R., Mukala P. (2026). V2X-JEPA: Self-Supervised Multi-Agent Joint Embedding Predictive Architecture for Robust Vehicle-to-Everything Perception. IEEE Internet Things J..

